# Health effects triggered by tritium: how do we get public understanding based on scientifically supported evidence?

**DOI:** 10.1093/jrr/rrab029

**Published:** 2021-04-28

**Authors:** Hideki Matsumoto, Yoshiya Shimada, Asako J Nakamura, Noriko Usami, Mitsuaki Ojima, Shizuko Kakinuma, Mikio Shimada, Masaaki Sunaoshi, Ryoichi Hirayama, Hiroshi Tauchi

**Affiliations:** Department of Experimental Radiology and Health Physics, University of Fukui School of Medical Sciences, Eiheiji-cho, Yoshida-gun, Fukui 910-1193, Japan; Institute for Environmental Sciences, Rokkasho-mura, Kamikita-gun, Aomori 039-3212, Japan; Department of Biological Sciences, Faculty of Science, Ibaraki University, 2-1-1 Bunkyo, Mito, Ibaraki 310-8512, Japan; Photon Factory, Institute of Materials Structure Science, High Energy Accelerator Research Organization (KEK), Tsukuba, Ibaraki 305-0801, Japan; Department of Environmental Health Sciences, Oita University of Nursing and Health Sciences, Oita 870-1201, Japan; Department of Radiation Effects Research, National Institute of Radiological Sciences (NIRS), National Institutes for Quantum and Radiological Science and Technology (QST), Chiba 263-8555, Japan; Laboratory for Advanced Nuclear Energy, Institute of Innovative Research, Tokyo Institute of Technology, 2-12-1, Oookayaka, Meguro-ku, Tokyo 152-8550, Japan; Department of Radiation Effects Research, National Institute of Radiological Sciences (NIRS), National Institutes for Quantum and Radiological Science and Technology (QST), Chiba 263-8555, Japan; Department of Charged Particle Therapy Research, National Institute of Radiological Sciences (NIRS), National Institutes for Quantum and Radiological Science and Technology (QST), Chiba 263-8555, Japan; Department of Biological Sciences, Faculty of Science, Ibaraki University, 2-1-1 Bunkyo, Mito, Ibaraki 310-8512, Japan

**Keywords:** tritium, health effects, radiation risk, tritiated water (HTO)

## Abstract

The Commission for ‘Corresponding to Radiation Disaster of the Japanese Radiation Research Society’ formulated a description of potential health effects triggered by tritium. This was in response to the issue of discharging water containing tritium filtered by the Advanced Liquid Processing System (ALPS), generated and stored in Fukushima Daiichi Nuclear Power Station after the accident. In this review article, the contents of the description, originally provided in Japanese, which gives clear and detailed explanation about potential health effects triggered by tritium based on reliable scientific evidence in an understandable way for the public, were summarized. Then, additional information about biochemical or environmental behavior of organically bound tritium (OBT) were summarized in order to help scientists who communicate with general public.

## INTRODUCTION

The Fukushima-Daiichi Nuclear Power Station was struck by a huge earthquake and tsunami on 11 March 2011. After the accident, the general public in Japan took a lively interest in the Advanced Liquid Processing System (ALPS)-filtered water containing tritium that was generated and stored in the Station, due to their concern about the health effects that may be triggered by tritium. Many reports about the health effects of incorporated tritium have been published [1]; however, these reports are mostly specialized for professional readership, and not intended for the general public. Here, the Commission for ‘Corresponding to Radiation Disaster of the Japanese Radiation Research Society’ drew up a description about health effects that may be triggered by tritium based on reliable scientific evidence and in a manner that can be easily understood by the general public, in a nod to the issue of discharging ALPS-decontaminated water containing tritium, generated and stored in Fukushima Daiichi Nuclear Power Station. In this review article, the contents of the description, which was originally prepared in Japanese (https://www.jrrs.org/assets/file/tritium_20191111.pdf), were translated and summarized, then additional information about biological or environmental behavior of organically bound tritium (OBT) were provided. The description gives a clear and detailed explanation about potential health effects triggered by tritium based on reliable scientific evidence in a way that is understandable to the public:

(i) What is tritium? (in terms of chemical substance)
(ii) What is tritium? (in terms of radioactive substance)
(iii) What happens in a human body after exposure to ionizing radiation?
(iv) Exposure pathways (inhalation, absorption and ingestion) and metabolism of tritium
(v) Health effects triggered by tritium.

Then, additional information that may help scientists are provided as follows:

(vi) Behavior of OBT in the environment
(vii) Academic perspectives.

The purpose of this document translated into English is not only for the sake of scientific understanding by the general public worldwide, but also to help scientists who face any scientific communication with public outreach and education concerning the health effects of radiation. Thus, we believe that the present review will be helpful to both scientists and the general public.

## What is tritium? (in terms of chemical substance)

**Fig. 1.**
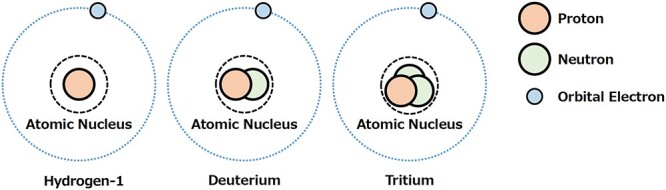
Hydrogen-1 and its isotopes.

Tritium is a rare, naturally occurring radioactive isotope of hydrogen. It was discovered by M. Oliphant (1901–2000) in 1934 [2]. In nature, the overwhelming majority of hydrogen atoms (over 99.9%) contain only a proton in their nucleus (^1^H). Deuterium (^2^H), another isotope of hydrogen that contains a proton and a neutron in the atomic nucleus makes up 0.0115%. The rest is tritium (^3^H), containing one proton and two neutrons in the atomic nucleus (Fig. 1). Hydrogen-1 and deuterium are stable isotopes of hydrogenic atoms, while tritium is radioactive.

Tritium undergoes β^−^ decay by emitting radiation in the form of low energy β-rays. It is naturally generated by the reactions of high energy neutrons and protons from cosmic rays in space with oxygen and nitrogen in the atmosphere. The number of tritium atoms generated naturally is 0.2 to 1/sec/cm^2^ in the earth’s surface area [3], i.e. the total number of tritium atoms generated for 1 sec is 1 to 5 × 10^18^, with the earth’s surface area being 5 × 10^14^ m^2^. Therefore, the total number of tritium atoms generated per year is 3.2 × 10^25^ to 1.6 × 10^26^, corresponding to 5.7 × 10^16^ to 2.9 × 10^17^ Bq (the becquerel being the activity of a quantity of radioactive material in which one nucleus decays per second). The concentration of tritium in the atmosphere is estimated to be about 13 mBq/m^3^ although it may vary with latitude [4].

Tritium is also artificially generated by nuclear fission reaction as a by-product of nuclear weapons tests and nuclear power stations. In these situations, tritium is either discharged to the sea or the atmosphere. A large amount of tritium in the atmosphere has its origin from nuclear weapons tests, especially hydrogen bomb tests between 1945 and 1984. The sum of radioactivity of tritium generated by nuclear weapons tests is estimated to be up to 1.9 × 10^20^ Bq [5].

**Fig. 2.**
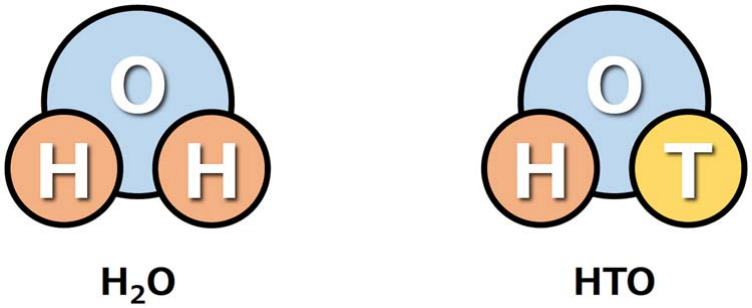
Molecules of water and tritiated water.

Tritium generated naturally and artificially reacts immediately with oxygen in the atmosphere, and the reaction products are brought into atmospheric/hydrological circulation as tritiated water (HTO) (Fig. 2).

## What is tritium? (in terms of radioactive substance)

**Fig. 3.**
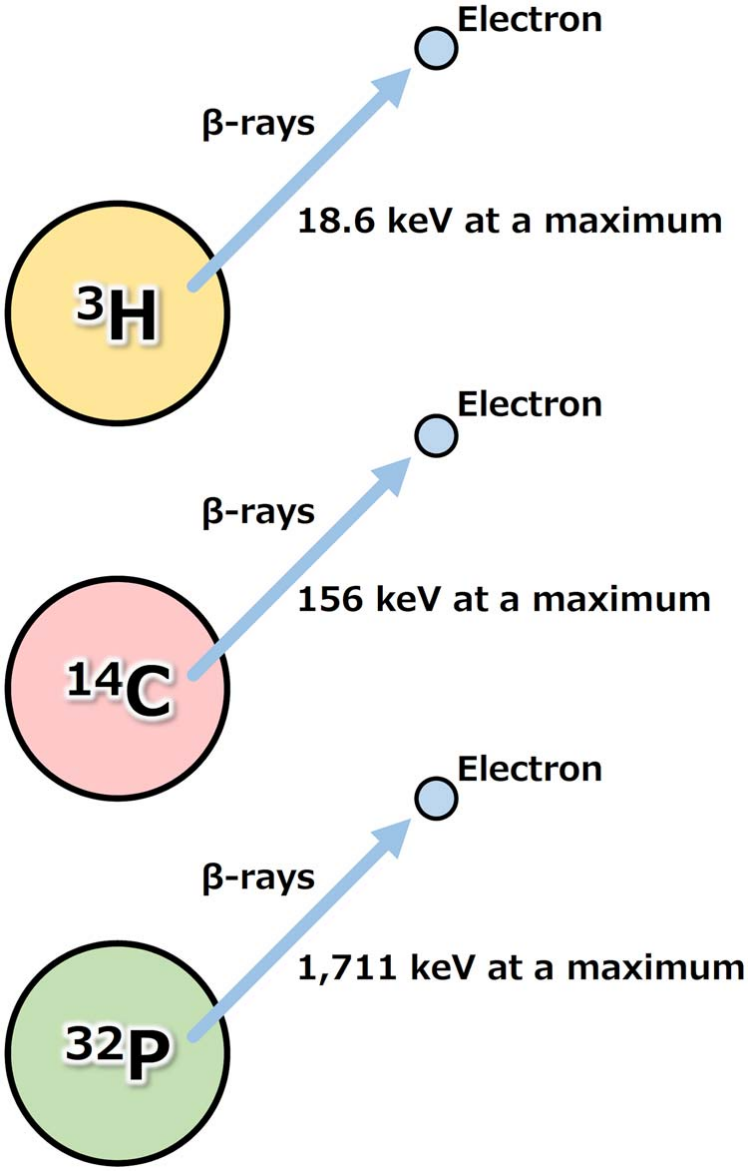
Energy of β-rays from tritium, carbon-14 and phosphorus-32.

The physical half-life of tritium is 12.3 years [6], and the radioactivity of tritium per 1 g in weight is 3.6 × 10^14^ Bq. As stated above, tritium undergoes β^−^ decay by emitting β-rays (electrons) with a maximum energy of 18.6 keV (5.7 keV on average). By comparison, carbon-14 (^14^C), which is frequently used in archaeological age determinations of animal and plant fossils, undergoes β^−^ decay by emitting β-rays with 156 keV at a maximum. Phosphorus-32 (^32^P), which is frequently used in biochemical experiments, undergoes β^−^ decay with emitting β-rays with 1,711 keV at a maximum. Thus, the energy of β-rays from tritium is quite low (Fig. 3), so that the range of the emitted rays is extremely short, 0.56 μm on average and 6 μm at a maximum, indicating that β-rays from external tritium will not likely traverse a nucleus (approximately 10 μm in diameter) of an animal cell. Therefore, in exposure to β-rays from tritium, we must consider an internal exposure due to inhalation, absorption and ingestion of tritium-containing chemicals such as HTO, rather than external exposure.

## What happens in a human body after exposure to ionizing radiation?

Health effects in humans triggered by ionizing radiation are classified into two groups: deterministic and stochastic effects.

The deterministic effects are health effects that displayed symptoms due to the killing of tissue stem cells in those exposed to ionizing radiation at more than threshold doses for tissue reactions. The threshold dose for tissue reactions is defined as a dose to induce tissue injury at the level of 1% incidence [7]. Typical early effects resulting in symptoms appearing over several weeks after exposure to ionizing radiation, are acomia and permanent infertility, as well as skin lesions and hematopoietic disorders. Cataracts are a typical late effect with symptoms arising after a long latent period extending to decades after exposure to ionizing radiation. The threshold doses for acomia, permanent infertility and cataracts are 3, 2.5–6, and 0.5 Gy delivered to the whole body, respectively. When pregnant women are exposed to ionizing radiation, embryonic death and malformation are the deterministic effects, which are provoked in fetuses. The threshold doses for both are 0.1 Gy as whole body exposure dose (0.1 Sv, here, the sievert [Sv] is a unit of radiation dose used for radiation protection to assess the health risk on humans), which is the minimal threshold dose among the various deterministic effects. On the other hand, the stochastic effects are health effects displayed stochastically by accumulating DNA mutations in cells of the tissues exposed to ionizing radiation. Typical stochastic effects are solid cancer and leukemia. Therefore, health effects provoked by ionizing radiation at below 0.1 Gy as a whole body exposure dose (0.1 Sv) are only the stochastic effects. There is still no evidence, however, for the stochastic effects provoked by whole body exposure to ionizing radiation of less than 0.1 Gy (0.1 Sv).

Ionizing radiation interacts with molecular components of the living organisms, such as DNA, proteins and lipids, and changes their functions. In particular, the chemical changes in DNA, which encodes genetic information, is important when considering the biological effects of ionizing radiation. It can induce DNA damage, such as strand breaks and structural alterations. When DNA damage is left unrepaired or incorrectly repaired, mutations and genome instabilities can be induced, which may be a cause of cancer. However, the DNA damage repair systems existing in human cells are consistently repairing DNA lesions induced either endogenously by metabolism or exogenously by environmental factors such as ionizing radiation. Two repair systems function mainly for DNA double strand breaks, which if not repaired can also result in cell death. One is non-homologous end joining (NHEJ) repair, which joins together broken ends of DNA, and the other is homologous recombination (HR) repair, which reconstructs DNA using undamaged DNA strands as a template. Some DNA sequences can be lost when DNA double strand breaks are repaired by the NHEJ pathway. In the human genome, however, the coding regions for proteins are only 2% of DNA, in other words, the loss of some sequences in the genomic DNA rarely leads to biological change. So it is generally regarded that biological changes are hardly induced, even if some DNA sequences are lost by the NHEJ pathway. In the HR pathway, DNA double strand breaks are repaired without loss of genetic information. However, the phase of cell cycles in which HR can function is restricted to specific phases (S and G2); in these phases, HR uses the undamaged sister chromatid DNA as a template to repair the damaged strand. Therefore, the HR pathway is available only in cells that are actively dividing [8]. As many protein molecules associated with these DNA repair systems possess individual functions, such as DNA damage recognition, signal transduction to other proteins and joining broken ends of DNA, it is elucidated that their cellular content and localization are changing by the minute in cells after exposure to ionizing radiation [9, 10].

## Exposure pathways (inhalation, absorption and ingestion) and metabolism of tritium

We have to follow the internal exposure to comprehend the health effects triggered by tritium. The exposure pathways of tritium are conceivable as follows;

(i) Inhalation of HTO in the atmosphere through the nose and mouth
(ii) Absorption of HTO through skin
(iii) Ingestion of HTO (or tritium-containing organic compounds) in food and drink.

**Fig. 4.**
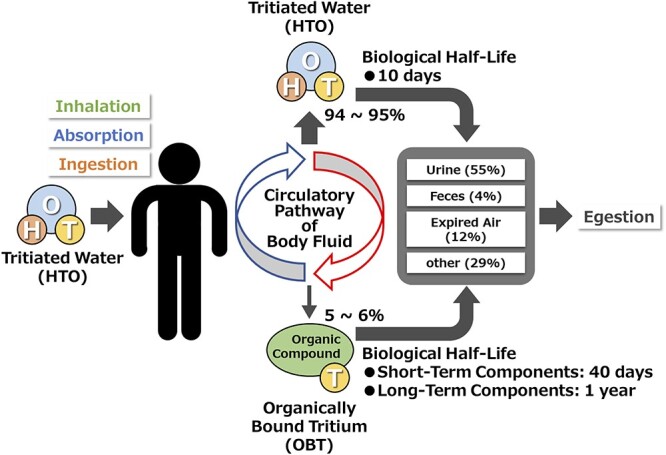
Metabolism of tritiated water in the human body [1, 11].

HTO that get incorporated into the body penetrates into the circulatory pathway of body fluids and is finally ejected from the body. The biological half-life of tritium is about 10 days because HTO incorporated into the body is ejected relatively quickly, similar to H_2_O [1]. To understand the effects of internal exposures by tritium, however, it is important to realize that a part of tritium atoms (5–6% of HTO absorbed into the body) exists as a component of the body due to exchange with hydrogen atoms in organic compounds such as proteins and carbohydrates in the body, the so-called OBT. OBT, especially tritium bound to carbon atoms in organic compounds remains longer in the body, because such OBT is difficult to exchange for other atoms in organic compounds. Thus, the biological half-life of OBT is about 40 days for a short-term component and about one year for a long-term component. Thus, as described above, most of the HTO absorbed into the body (94–95%) will be ejected relatively quickly. Exposure pathway (inhalation, absorption and ingestion) and metabolism of HTO incorporated into the body is drawn in Fig. 4, according to UNSCEAR [1] and ICRP [11].

## Health effects triggered by tritium

The latest committed effective dose coefficient of tritium incorporated into the body via the oral route in adults is 1.9 × 10^−8^ mSv/Bq for the soluble form, and that of the biogenic form is 5.1 × 10^−8^ mSv/Bq [12]. Age-dependent coefficient values for the general public, which are described in ICRP [11], are summarized in Table 1. These values are about 1/300th of that of cesium-137 (^137^Cs) (1.3 × 10^−5^ mSv/Bq), suggesting that health effects triggered by tritium are extensively low compared with ^137^Cs under the same uptake amount of Bq (Table 1).

**Table 1:** Committed effective dose coefficient [11] of tritiated water, cesium-134, cesium-137 and iodine-131 (mSv/Bq)

	Tritiated water	Cesium-134	Cesium-137	Iodine-131
Baby (3 months)	6.4 × 10^−8^	2.6 × 10^−5^	2.1 × 10^−5^	4.8 × 10^−5^
Child (1 year)	4.8 × 10^−8^	1.6 × 10^−5^	1.2 × 10^−5^	1.8 × 10^−5^
Child (5 years)	3.1 × 10^−8^	1.3 × 10^−5^	9.6 × 10^−6^	1.0 × 10^−5^
Child (10 years)	2.3 × 10^−8^	1.4 × 10^−5^	1.0 × 10^−5^	5.2 × 10^−5^
Child (15 years)	1.8 × 10^−8^	1.9 × 10^−5^	1.3 × 10^−5^	3.4 × 10^−5^
Adult	1.8 × 10^−8^	1.9 × 10^−5^	1.3 × 10^−5^	2.2 × 10^−5^

### Effects triggered by tritium on individual death

Brue et al. analyzed a half lethal dose within 30 days (LD_50/30_) in CF1 female mice who were administered with HTO intraperitoneally at 1.3 × 10^8^ to 8.4 × 10^9^ Bq. As a result, the LD_50/30_ was 9 Gy, corresponding to 3.7 × 10^7^ Bq/g body weight [13]. According to subsequent reports, LD_50/30_ was 8 Gy in CF1 female mice, corresponding to 3.3 × 10^7^ Bq/g body weight [14], and LD_50/30_ was 13 Gy in (C57BL/6 N × C3H/He) F1 female mice, corresponding to 4.7 × 10^7^ Bq/g body weight [15]. Therefore, it is conceivable that the LD_50/30_ in mice administered intraperitoneally HTO is around 10 Gy corresponding to about 4.0 × 10^7^ Bq/g body weight.

There have been two radiation exposure accident reports in human, due to long-term ingestion of tritium, which occurred at two watch factories in Europe in the 1960s. At that time, luminous paints containing tritium were commonly used to draw the face of a watch. In one case, a factory worker ingested tritium integrated into luminous paints over 7.4 years. His exposure dose was estimated at 3–6 Sv based on the content of tritium in the urine. He developed isochromic anemia, and subsequently died of pancytopenia [16]. In the second case, a factory worker who ingested tritium integrated into luminous paints over three years, with an estimated accumulated dose of 10–20 Sv, died of pancytopenia after following a similar disease course [15]. It should be noted that these two people were also exposed to radioisotopes other than tritium over a long period [17].

### Effects triggered by tritium on carcinogenesis

There are currently no data on cancer risk of tritium for humans. However, several findings were obtained in experiments using mice. In one experiment approximately 550 F1 female mice (C57BL/6 N × C3H/He) ingested HTO continuously at various concentrations for their entire lifetime, their average lifespan, population bearing cancer, and types of cancers were analyzed [18, 19]. The exposure mean dose-rate (mGy/day) was estimated from the content of tritium in some organs harvested after more than seven days from the onset of the experiment since the concentrations of tritium in the body achieved equilibrium in seven days.

**Fig. 5.**
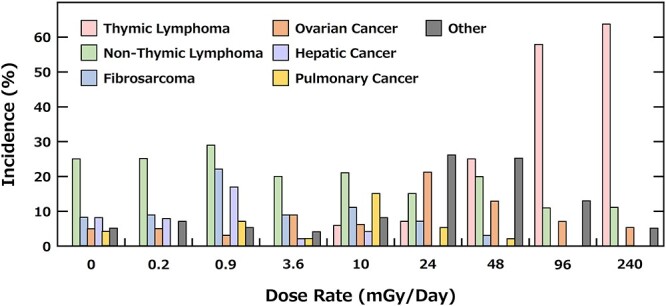
The types of cancers developing in mice ingested HTO [18].

**Fig. 6.**
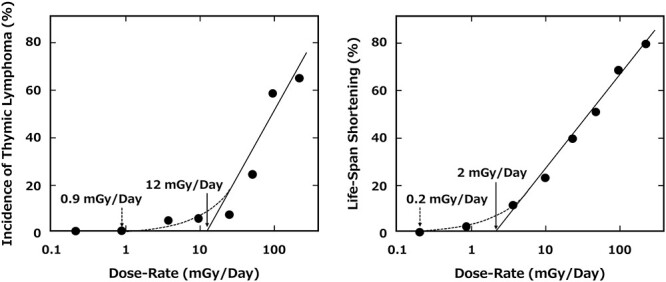
Incidence of thymic lymphoma and lifespan shortening in mice ingested at different dose-rate of HTO [19].

Half of the control mice developed cancer, but an obvious increase in cancer induction was found in mice that ingested HTO at the concentration more than 10 mGy/day. These data suggested that a dose below 3.6 mG/day, had no observable effect on cancer incidence. Furthermore, the types of cancers ad the frequency of their development in mice that ingested HTO at concentrations lower than 3.6 mGy/day was similar to those in the control mice (Fig. 5) [18]. The average lifespan of mice that ingested HTO at less than 3.6 mGy/day, corresponding to 1.4 × 10^8^ Bq/liter of HTO for the entire lifetime, differed little from that of the control mice (Fig. 6). The average lifespan of mice that ingested HTO at more than 10 mGy/day shortened with increasing HTO concentrations. The authors further analyzed the relationship of life-shortening and incidence of thymic lymphoma with dose rate. They proposed two types of threshold dose rates, one is ‘essential dose-rate threshold’ of 2 mGy/day for life-shortening and that of 12 mGy/day for thymic lymphoma, and the second is ‘practical tail threshold’ of 0.2 mGy/day for life-shortening and that of 0.9 mGy/day for carcinogenesis (Fig. 6, Table 2) [19]. Collectively, it is conceivable that the practical threshold of exposure mean dose-rate for carcinogenesis induced by HTO range from 0.9 and 12 mGy/day. This means that an incidence of cancer in mice ingesting 3.5 × 10^8^ Bq/liter of HTO for their entire lifetime is almost the same to that of the control mice. It should be noted that these findings are from experiments on mice, and the findings in humans are still unclear.

**Table 2:** Population bearing cancer and average lifespan in mice ingested at different dose-rate of HTO [19]

Dose Rate (mGy/Day)	0	0.2	0.9	3.6	10	24	48	96	240
Population Bearing Cancer (%)	52	49	78	46	83	70	70	84	76
Average Lifespan (Day)	808	790	758	804	622	481	414	259	165

### Effects triggered by tritium on the cerebral nervous system

Gao et al. analyzed the cerebral nervous system in fetal Wistar rats exposed at 46, 92 and 273 mGy in the uterus of female rats ingesting HTO on the thirteenth day of pregnancy. The weight of brains in infant rats at the age of 45 or 46 days was significantly decreased in the group exposed at 273 mGy. Disabilities in learning and recollection with decreasing a cellular density of spindle nerve cells in the hippocampus were found in infant rats exposed at 92 mGy [20].

Cognitive function deficit was found in infant C57BL/6 J mice exposed to 100 or 300 mGy in the uterus of animals administrated HTO into the peritoneal cavity [21].

## Behavior of organically bound tritium in environment

Because some people are concerned about biological accumulation of OBT in the environment, we briefly summarize the behavior of OBT in the ecosystem. Scientific data about the environmental behavior of OBT are still limited, however, it is clear that biological accumulation is not the case for tritium including OBT. Here, we introduce the summary of published review articles about OBT the in biosphere [22].

OBT in the environment can be classified into the exchangeable OBT (E-OBT) and non-exchangeable OBT (NE-OBT). In grass plants, it is estimated that about 65–70% of OBT is NE-OBT and the rest is E-OBT. The E-OBT in living organisms easily equilibrates with the tissue free water tritium (TFWT) and can be exceeded into surrounding environment. In contrast, the NE-OBT persists for a long time in plants, microbes in soil and in fish. Therefore, NE-OBT in living organism may reflect the historical (from several months to several years) concentration of tritium in the environment. As such, there has been report that tritium concentration in algae or fish are higher than the surrounding water at the study site [22]. It should be noted that the OBT in lichens or timbers may be a good indicator of historical concentration of tritium in the environment for a longer period (several years) although the methodology for the analysis of those environmental tritium is not precisely established.

OBT in the environment may be transferred into the food chain, including that of humans. Many reports suggest that around 70% of OBT could be sequestered in a manner that is difficult to exchange with TFWT or the surrounding environment [22]. They function as reservoirs of OBT in the terrestrial biosphere, although the residence time is shorter than physical radioactive decay of tritium (12.3 y). These observations indicate that over half of OBT is kept recycled in the food chain when a large amount of tritium is introduced from any artificial source such as atomic weapons. Thus, keeping tritium concentration in the terrestrial environment low where radiation risks are negligible is important for maintaining human health.

## Academic perspectives

We summarized here the latest knowledge about biological effects of tritium, both the inorganic- and organic-bound chemical structure. We believe that the present review would be a great help to scientists who are engaged in public education on the health effects of tritium. One should recognize the relationship between the dose of tritium exposure and the resulting risk based on scientific evidence. However, the biological effects of low dose or low-dose rate radiation remain controversial regardless of the types of radiations. Thus, we need uniquely sensitive experimental systems to evaluate the biological effects triggered by tritium, because the biological effects of HTO are mainly due to low dose/low-dose rate exposure. Unfortunately, there are few such systems available at present. In addition, as most of the scientific evidence of the biological effects triggered by tritium was obtained using model animals, the possibility exists that we may not be able to accurately extrapolate the risk to humans. We hope strongly that the examination and elucidation of the health effects triggered by exposure to low dose/low-dose rate ionizing radiation will continuous into the future in order to secure public health.
